# Supplementary material for: Venomix: a simple bioinformatic pipeline for identifying and characterizing toxin gene candidates from transcriptomic data
Source: PeerJ. 2018 Jul 31;6:e5361. doi: 10.7717/peerj.5361 (PMC6074769; doi:10.7717/peerj.5361)
Supplement: Supplemental Information 3 [file peerj-06-5361-s003.gz › FinalOutput_E-6/Kunitz-type_serine_protease_inhibitor_homolog_delta-dendrotoxin_2/finaltree.pdf]

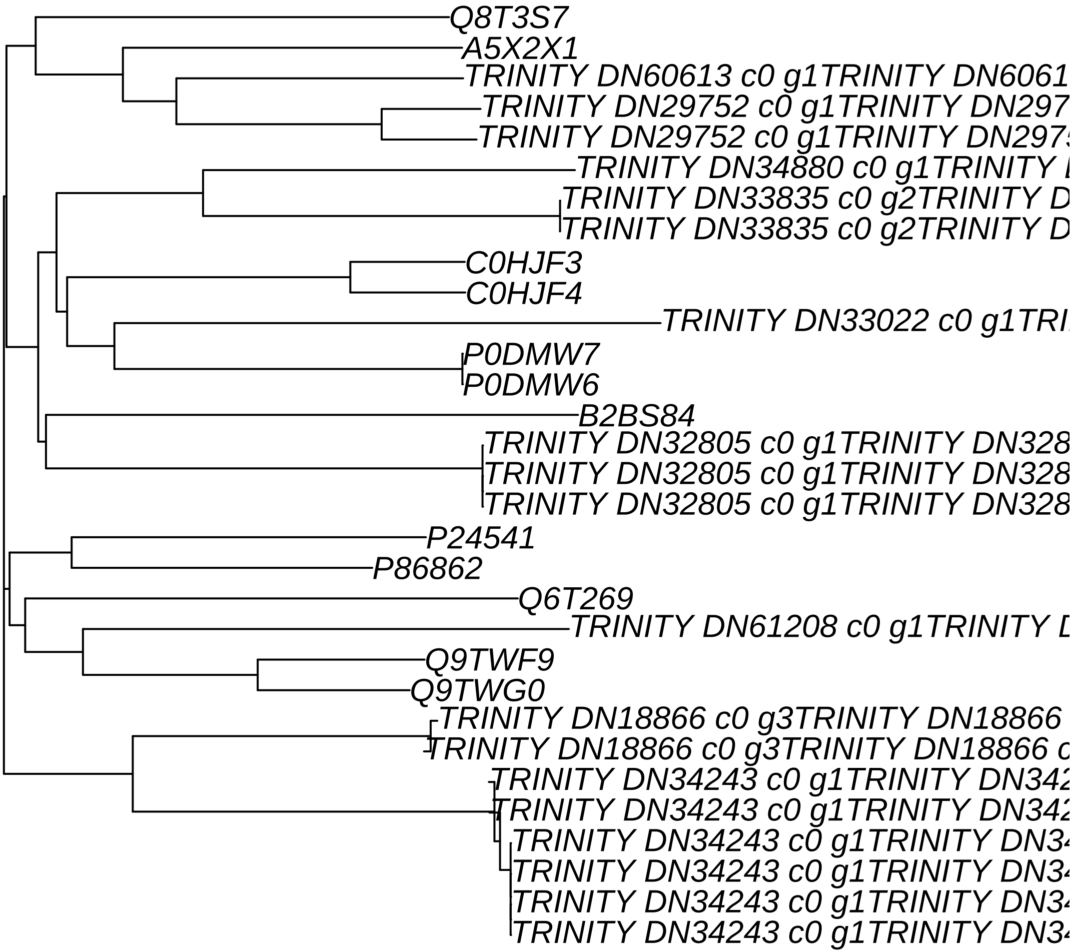
Q8T3S7
A5X2X1
TRINITY DN60613 c0 g1TRINITY DN6061
TRINITY DN29752 c0 g1TRINITY DN297
TRINITY DN29752 c0 g1TRINITY DN297
TRINITY DN34880 c0 g1TRINITY
TRINITY DN33835 c0 g2TRINITY D
TRINITY DN33835 c0 g2TRINITY D
C0HJF3
C0HJF4
TRINITY DN33022 c0 g1TRI
P0DMW7
P0DMW6
B2BS84
TRINITY DN32805 c0 g1TRINITY DN328
TRINITY DN32805 c0 g1TRINITY DN328
TRINITY DN32805 c0 g1TRINITY DN328
P24541
P86862
Q6T269
TRINITY DN61208 c0 g1TRINITY
Q9TWF9
Q9TWG0
TRINITY DN18866 c0 g3TRINITY DN18866
TRINITY DN18866 c0 g3TRINITY DN18866
TRINITY DN34243 c0 g1TRINITY DN342
TRINITY DN34243 c0 g1TRINITY DN342
TRINITY DN34243 c0 g1TRINITY DN3
TRINITY DN34243 c0 g1TRINITY DN3
TRINITY DN34243 c0 g1TRINITY DN3
TRINITY DN34243 c0 g1TRINITY DN3
